# The Relationship between the HLA-G Polymorphism and sHLA-G Levels in Parental Pairs with High-Risk Pregnancy

**DOI:** 10.3390/ijerph16091546

**Published:** 2019-05-01

**Authors:** Olimpia Sipak, Aleksandra Rył, Anna Grzywacz, Maria Laszczyńska, Małgorzata Zimny, Beata Karakiewicz, Iwona Rotter, Danuta Kosik-Bogacka, Cezary Cybulski

**Affiliations:** 1 Department of Obstetrics and Pathology of Pregnancy, Pomeranian Medical University in Szczecin, 71-210 Szczecin, Poland; mmzimny@wp.pl; 2 Department of Medical Rehabilitation and Clinical Physiotherapy, Pomeranian Medical University in Szczecin, 71-210 Szczecin, Poland; aleksandra.ryl@pum.edu.pl (A.R.); iwrot@wp.pl (I.R.); 3 Independent Laboratory of Health Promotion, Pomeranian Medical University in Szczecin, 70-103 Szczecin, Poland; anna.grzywacz@pum.edu.pl; 4 Department of Histology and Developmental Biology, Pomeranian Medical University in Szczecin, 71-210 Szczecin, Poland; laszcz@pum.edu.pl; 5 Department of Public Health, Pomeranian Medical University in Szczecin, 71-210 Szczecin, Poland; beata.karakiewicz@pum.edu.pl; 6 Independent of Pharmaceutical Botany, Department of Biology and Medical Parasitology, Pomeranian Medical University in Szczecin, Powstańców Wielkopolskich 72, 70-111 Szczecin, Poland; kodan@pum.edu.pl; 7 Department of Genetics and Pathology, Pomeranian Medical University in Szczecin, 71-252 Szczecin, Poland; cezarycy@pum.edu.pl

**Keywords:** HLA-G, allele, complicated pregnancy

## Abstract

Human leukocyte antigen G (HLA-G) is observed in immune system cells and other organs. It is a class Ib molecule, which plays a pivotal role in the implantation and maintenance of pregnancy. The aim of this study was to assess the relationship between serum sHLA-G levels and the HLA-G allele in parental pairs with complicated obstetric histories. The clinical material consisted of 210 women and 190 men with the experience of a complicated or an unsuccessful pregnancy. The control group included parents―89 women and 86 men―lacking complicated obstetric histories. We applied genetic analysis methods: isolation of genomic DNA, sequencing, and determination of serum sHLA-G levels. There were no statistically significant differences in the frequencies of the HLA-G −725 C>G polymorphism between particular experimental groups compared with the control group (*p* > 0.05). The median sHLA-G levels in the women with the HLA-G10101 allele (15.4 U/mL) were significantly higher than in the women with other alleles (*p* < 0.05). The HLA-G 10101 allele seems to protect against antiphospholipid syndrome, which may be associated with increased serum sHLA-G levels in its carriers. The relationship between serum sHLA-G levels and the HLA-G polymorphisms in the Polish population requires further investigation.

## 1. Introduction

Human leukocyte antigen-G (HLA-G) is a nonclassical HLA class Ib molecule. This protein, which is generated via alternative splicing, has four isoforms associated with the cell membrane (G1, G2, G3, and G4) and three soluble isoforms (G5, G6, and G7). During pregnancy, the maternal immune system must tolerate antigenically alien ovum cells, whereas a fetus developing in the uterine cavity shows an expression of antigens coming from both parents. A healthy pregnancy is believed to be dependent on many mechanisms, including weak expression of classic major histocompatibility complex (MHC) class I antigens [1].

HLA-G is observed in immune system cells and in other organs, such as the uterus, thymus, ovaries, and placenta [2,3,4,5]. The immune-regulatory properties of HLA-G have been well-documented [5]. These molecules can be observed on the surface of villous cytotrophoblasts in the first trimester of pregnancy and on the surface of extravillous trophoblasts [6]. HLA-G molecules are believed to inhibit the activity of natural killers (NKs) and cytotoxic T lymphocytes in the decidua and, thus, protect fetal cells against cytolysis [7,8].

A soluble HLA-G protein appears to be present in women at all stages of pregnancy. It has been detected in amniotic fluid and also in umbilical cord blood [9,10,11]. It disturbs the function of T CD8+ lymphocytes, which act against the father’s antigens, and induces their apoptosis [1]. There are also reports suggesting that HLA-G is capable of presenting endogenous viral antigens to the mother’s T lymphocytes [12] and of stimulating the mother’s lymphocytes to produce cytokines. 

The aim of this study was to assess the relationship between the *HLA-G* allele and serum sHLA-G levels in women with pregnancies complicated by antiphospholipid syndrome (APS), preeclampsia (PE), intrauterine growth restriction (IUGR), and recurrent spontaneous abortion (RSA).

## 2. Material and Methods

### 2.1. Experimental and Control Groups

The clinical material consisted of retrospectively assessed pairs of parents (210 women aged 20–35 years, and 190 men aged 21–42 years) who had experienced a complicated or an unsuccessful pregnancy. The control group comprised parents (89 women and their 86 partners) whose offspring had been born without any complications during pregnancy. From each participant, a 9 mL peripheral blood sample was taken from a cubital vein to determine the HLA-G allele and its polymorphisms.

All women included in the study were patients of the Clinic of Maternal and Fetal Medicine, the Outpatient Clinic at the Clinic of Maternal and Fetal Medicine, and the Rheumatology Outpatient Clinic, Pomeranian Medical University in Szczecin (Poland). The protocol was approved by the Bioethical Commission of (covered for blind review) (approval number BN-001/94/07). All participants gave voluntary informed written consent to take part in the study. 

### 2.2. Division into Groups

Besides the control group (C), the subjects of this study (women and their partners) were split into four experimental groups: one with antiphospholipid syndrome, one with preeclampsia, one with intrauterine growth restriction, and one with recurrent spontaneous abortion. The following criteria were employed:

1. Antiphospholipid syndrome (affecting 70 women with 54 partners) was diagnosed using laboratory and clinical criteria (history of autoimmune diseases, thrombotic complications, and embolic complications, as well as general obstetric history). Inclusion criteria were [13]: (1) thrombosis (meaning at least one episode of capillary thrombosis in any part of the body, venous thrombosis, or arterial thrombosis—excluding superficial venous thrombosis—as confirmed by histological examination or imaging); (2) obstetric failure (meaning at least one premature birth of a morphologically normal fetus prior to week 34 of pregnancy because of placental insufficiency, eclampsia, or preeclampsia; severe death of at least one morphologically normal fetus after week 10; or at least three spontaneous miscarriages prior to week 10 from other than anatomical causes, hormonal disorders of the mother, or chromosomal disorders of both parents); (3) laboratory criteria—specifically, lupus anticoagulant—detected in plasma at least twice (minimum of twelve weeks apart) through methods recommended by the International Society on Thrombosis and Haemostasis; mean or high levels of IgG or IgM class anticardiolipin antibodies (>40 GPL or MPL or <99th percentile) detected on at least two occasions minimum 12 weeks apart by a standardized ELISA method; or anti-β2-glycoprotein I antibodies in serum or plasma measured over the 99th percentile at least twice (minimum of twelve weeks apart).
2. Severe preeclampsia (affecting 43 women with 43 partners) was diagnosed by [14]: (1) proteinuria and higher blood pressure after week 20 of pregnancy (except in cases of multifetal pregnancy or gestational trophoblastic disease) and; (2) no proteinuria in cases where the following happened for the first time after week 20: disorders of the vision, pulmonary edema, central nervous system disorders, liver disease (doubled transaminase activity), impaired renal function (creatinine over 1.1 mg/dL or doubling in creatinine in the absence of a history of kidney disease), or thrombocytopenia (blood platelet count below 100,000 per µL).
3. Intrauterine growth restriction (affecting 58 women with their 58 partners) involved a fetal weight measured at the first ultrasound at the 10th percentile or lower for the gestational age, excluding causes of intrauterine growth restriction, such as smoking, alcohol consumption, diabetes, hypertension, genetic determinants, preeclampsia, uterine abnormalities, infections, renal diseases, taking medicine, autoimmune diseases, and malformations of fetal developmental.
4. Recurrent spontaneous abortion (affecting 58 women with their 58 partners) involved having at least three spontaneous miscarriages in the first trimester, excluding other causes of miscarriage. Normal karyotype was determined for all couples, and health problems like thyroid and adrenal gland diseases, diabetes, infections (including herpes, syphilis, HIV, varicella, rubella, cytomegaloviral disease, toxoplasmosis, and others), anatomic abnormalities, and autoimmune diseases (presence of lupus anticoagulant, anticardiolipin antibodies, or antinuclear antibodies) were excluded.
5. The control group contained 89 healthy women with their 86 partners. These women lacked significant perinatal history and had given birth at least twice after uncomplicated pregnancies. Thrombotic and embolic complications, as well as concomitant autoimmune diseases, were excluded.

### 2.3. Isolation of Genomic DNA from Peripheral Blood Leukocytes

Genomic DNA was isolated using the detergent method, in which peripheral blood samples of 10 mL each were collected from patients. Each sample was used with IGEPAL detergent (Sigma), 1 mL of 10% edetate disodium, and 20 mL of TKM buffer (10 mM Tris-HC1, 2 mM EDTA, 10 mM KCI, and 4 mM MgC1_2_) to degrade the cell membranes. The samples were then spun for 10 min (at 12 °C and 3400 rpm). Then, 30 and 20 mL of TKM were added in stages to the leukocyte sediment before being spun a second time. Next, 2 mL of TKM was added in order to break the sediment and obtain a homogeneous suspension. A 0.5 mL amount of 10% SDS (sodium dodecyl sulphate) was added to the resulting pure leukocyte sediment before incubation in a water bath at 60 °C for 7 min; at this point, the protein junctions and cell membranes were broken. The protein was salted out with 1 mL of 5 M NaCl solution and shaken until a homogeneous emulsion was obtained. This was spun at 9500 rotations per minute and 12 °C before the addition of 5 mL of 96% ethanol to the supernatant in order to precipitate out the DNA. The DNA was finally rinsed out with 1 mL ethanol, dried in a vacuum centrifuge for 10 min, and 3–4 drops of TE buffer (10 mM Tris-HCl, 1 mM EDTA) were added. The isolated DNA was diluted to 50 ng/µL.

### 2.4. Sequencing

We sequenced exons 2, 3, and 4 of *HLA-G*. Preparative polymerase chain reaction (PCR) was performed in 25 mL of solution containing 50 ng of the genomic DNA, 2.5 µL 10× reaction buffer (Polgen, Łódź, Poland), 200 µM of each deoxynucleotide, 6 pM of each primer, and 1 U Taq DNA polymerase. Preparative PCR was performed in 35 cycles with an initial denaturation temperature of 95 °C for 5 min; denaturation then went ahead at 95 °C for 30 s; primers were annealed at 55–60 °C for 30–40 s; elongation conditions were 72 °C for 30 s; and finally, elongation was at 72 °C for 5 min.

The amplicons were put on a 100× Microcon column (Amicon, Darmstadt, Germany) in an Eppendorf tube. We added 400 µL distilled water and spun the assembly at 1850× *g* for 15 min. We decanted the filtrate and poured 400 µL of water over the reaction products remaining on the filter, spun again, and repeated this process three times in total. To retrieve the purified PCR products, the column was inserted into a new test tube and spun at 9000× *g* for 10 min. Twenty microliters of water were used to dilute the product. We carried out asymmetric PCR using an automatic Gene Amp PCR 9600 thermocycle system (Perkin Elmer, Waltham, MA, USA) with a reaction mixture of 30 pM primer, 4 µL of a purified PCR product, and 8 µL of Prism Ready Reaction Mix (DyeDeoxy Terminator Cycle Sequencing Kit, Applied Biosystems, Thermo Fisher Scientific, Waltham, Massachusetts, USA). The sequential PCR was carried out with the following parameters: initial denaturation at 96 °C for 15 s; 25 cycles of denaturation at 94 °C for 20 s; primer annealing at 55 °C for 30 s; and DNA elongation at 60 °C for 4 min.

The amplicons were precipitated with 96% ethanol, rinsed with 70% ethanol, and dried in an Eppendorf Concentrator 5301 vacuum apparatus. The amplicons were then dissolved in 4 µL loading buffer (consisting of 0.05% Dextran Blue, 150 µL deionized formamide, and 50 µL 50 mM EDTA). Denaturation of the samples occurred at 94 °C for 4 min. They were then put into an ice water bath and placed on denaturing polyacrylamide sequence gel (6% acrylamide: 19:1, 1× TBE, 8 M urea). Electrophoretic separation was carried out with a 373A DNA Sequencer (Applied Biosystems). Collection and analysis of the electrophoresis data employed the 373 Data Collection and Analysis software (Applied Biosystems).

The *HLA-G* alleles we observed in our study were determined by comparing the base pair sequences in exons 2, 3, and 4 with those of the *HLA-G* alleles available from the Nolan Institute’s website (https://www.anthonynolan.org/) [15]. 

### 2.5. Analysis of the −725 C>G Polymorphism in the HLA-G Promoter Region

The −725 C>G polymorphism in the HLA-G promoter region was determined by restriction fragment length polymorphism-polymerase chain reaction (RFLP-PCR). The particular fragments were amplified in a thermal cycler (Applied Biosystems GeneAmp PCR System 9700, Foster City, CA, USA). The following primers were used in PCR:

F 5′GAA AGT GAA ACT TAA GAG CTT TGT GAG GC 3′
R 5′TTG GTA ACC CCT GAA TGA TCA G 3′

A 25 µL sample of the reaction mixture contained 50 ng of the genomic DNA, 2.5 µL of 10× reaction buffer (Polgen, Łódź, Poland), 200 µM of each deoxynucleotide (ATP, CTP, GTP, and TTP), 6 pM of each primer, and 1 U Taq DNA polymerase. 

PCR was performed under the following conditions: preliminary denaturation (94 °C, 5 min); 33 cycles consisting of denaturation (94 °C, 25 s), primer annealing (50 °C, 35 s), and complementary DNA elongation (72 °C, 35 s). The amplicons were digested with the BstUI restriction enzyme (EURX, Gdańsk, Poland). Digestion was performed using 15 µL of the PCR product and 15 µL of digesting mixture containing 11.9 µL H_2_O, 3 µL 1× buffer 10× Medium (EURX), and 1 U of the BstUI restriction enzyme (EURX) that cut PCR products if the C allele was present. The amplicons were digested under a drop of mineral oil. Blind trials were performed. PCR products were digested with a specific restriction enzyme and subjected to electrophoresis (on 3% agarose gel). In selected cases (10% of all samples analyzed), the RFLP-PCR results were verified by sequencing analysis.

### 2.6. Determination of Serum sHLA-G Levels

Blood samples (3 mL) were collected from 175 women from the groups with antiphospholipid syndrome (*n* = 57), preeclampsia (*n* = 28), and intrauterine growth restriction (*n* = 18), and from the control group (*n* = 72) to determine sHLA-G levels. We applied the ELISA method using reagent kits from BioVendor Laboratory Medicine, Inc. (catalogue no. RD194070100, Brno, Czech Republic). The method’s sensitivity was 2 U/mL. The analysis was performed in the Research and Service Laboratory, Clinic of Maternal and Fetal Medicine, Pomeranian Medical University in Szczecin (Poland).

### 2.7. Statistical Analysis

Statistical analysis was performed using Statistica v. 17.0 (StatSoft, Inc., Tulsa, OK, USA). A chi-squared test was applied to verify whether genotype frequencies matched the Hardy–Weinberg equilibrium (H-W). Noncontinuous variables were assessed using Pearson’s chi-square test. The frequency of the −725 C>G polymorphism in the *HLA-G* promoter region was evaluated by Fisher’s two-sided exact test, and sHLA-G levels by the Mann–Whitney U test. The level of significance was set as *p* ≤ 0.05.

## 3. Results

### 3.1. Serum sHLA-G Levels in Women with Previous Complicated Pregnancies

The median of sHLA-G levels (Table 1) in the group of pregnant women lacking complicated obstetric histories was 12.3 U/mL. The median of sHLA-G levels in the women with the *HLA-G* 10101 allele (15.4 U/mL) was significantly higher than in those with other alleles (*p* < 0.05). The median of sHLA-G levels for the *HLA-G* 10102 allele was 10.75 U/mL. In the case of other alleles, the median sHLA-G levels were determined for small subgroups of women (*n* < 7).

The highest median sHLA-G level (Table 2) was observed in the women with intrauterine growth restriction (14.9 U/mL), and the lowest median sHLA-G level was noticed in the women who had experienced preeclampsia (10.1 U/mL). The differences in the median sHLA-G levels between the groups were statistically insignificant. 

### 3.2. Frequency of the HLA-G −725 C>G Polymorphism in Parental Pairs with Regard to Pregnancy Complications

The control group met the criteria for the Hardy–Weinberg equilibrium for the −725 C>G polymorphism (women and men *p* = 0.98). Linkage disequilibrium in the control group was statistically insignificant (women *r* = 0.05, *p* = 0.626; men *r* = 0.09, *p* = 0.377). Table 3 shows the frequency of the −725 C>G polymorphism in the *HLA-G* promoter region in the parental pairs with antiphospholipid syndrome, preeclampsia, intrauterine growth restriction, and recurrent spontaneous abortion, and in the control group. There were no statistically significant differences in the frequencies of the *HLA-G* −725 C>G polymorphism between particular experimental groups compared with the control group (*p* > 0.05).

No statistical significance (*p* > 0.05) was demonstrated by analysis of the frequency of the alleles of the −725 C>G 3′UTR polymorphism in the *HLA-G* gene shared by both parents in the pairs with complicated pregnancies (Table 4).

## 4. Discussion

During pregnancy, maternal immunological tolerance of the semiallogeneic fetus and the placenta results from the activity of HLA-G. High HLA-G expression in trophoblast cells suggests that this relationship may play a role in the immune response in areas where maternal and fetal tissues come into contact [15]. Studies have provided support for the notion that sHLA-G measurement during pregnancy is of prognostic value, and the presence of this antigen in pregnancy seems to be essential for implantation and further development of embryos [16].

Our study demonstrated that the nonpregnant women and the women with various pregnancy complications had different serum sHLA-G levels; however, the differences were not statistically significant. Nevertheless, it is worth emphasizing that the level of sHLA-G in women with past preeclampsia was lower than in the control group.

Similar research was carried out by Mubarak et al. [17], who analyzed sHLA-G levels in women after spontaneous abortion, women after uncomplicated pregnancies and vaginal delivery, nonpregnant women, and men. The authors demonstrated that the women after spontaneous abortion had higher sHLA-G levels than those who had given birth vaginally and the nonpregnant women. They also found that in the second trimester sHLA-G levels were higher than in the first trimester in all studied pregnant women.

It is worth emphasizing that the levels of sHLA-G are also affected by some diseases experienced by women during pregnancy. D’Almeida et al. [18] reported that the mothers infected with placental malaria were more likely to give birth to babies with high sHLA-G levels. They found that the level of sHLA-G in pregnancy may be related to immune tolerance associated with placental malaria. In another study [19,20,21,22], decreased HLA-G levels in the urine of women correlated with pregnancy disorders, such as recurrent spontaneous abortion and preeclampsia. Steinborn et al. [23] reported that women with sHLA-G levels below 9.95 ng/mL were at a greater risk for placental abruption during pregnancy than their healthy counterparts. Rebmann et al. [24], on the other hand, claimed that the *HLA-G* alleles can be divided into those that determine high and low risk of complications. There are also publications denying the relationship between sHLA-G levels and complications, such as spontaneous abortion [25,26] and preeclampsia [27,28].

Many single nucleotide polymorphisms (SNPs) in the *HLA-G* promoter region have been identified and described. Aside from the −725 (G/C/T) mutation analyzed in our study, those functionally active also include the −201 (A/G) and the −964 (G/A) SNPs. It has been demonstrated that the substitution for guanine at the −725 position correlated with increased *HLA-G* expression [29]. Our study did not reveal any relationships between the −725 C>G polymorphism and pregnancy complications. 

According to Ober et al. [29], parental pairs where both partners have the −725 G allele are more likely to have recurrent spontaneous abortion than pairs without this allele. However, in our research such a relationship in the pairs with complicated pregnancies was not noted. Similarly, it was not observed by Porras et al. [30], who analyzed the link between the *HLA-G* −725C>G (rs1233334) polymorphism and recurrent spontaneous abortion in Mexican women, that this polymorphism did not contribute to the risk of miscarriage. Xue et al. [31], on the other hand, demonstrated that recurrent spontaneous abortions in the Chinese population were visibly more common among heterozygotes with the ins/del 14 bp in the *HLA-G* 3′UTR region. In a study of Indian women, alleles with multiple 14 bp were more common in those who had experienced recurrent spontaneous abortion than in those with uncomplicated pregnancies [32]. 

## 5. Conclusions

Based on our research, the *HLA-G* −725 C>G polymorphism likely has no impact on the risk of pregnancy complications, such as preeclampsia, intrauterine growth restriction, antiphospholipid syndrome, and recurrent spontaneous abortion. Nonetheless, the *HLA-G* 10101 allele seems to protect against antiphospholipid syndrome, which may be associated with increased serum sHLA-G levels in its carriers. The relationship between serum sHLA-G levels and *HLA-G* polymorphisms in the Polish population requires further investigation.

## Figures and Tables

**Table 1:** The relationship between median (Me), minimum (min), and maximum (max) serum sHLA-G levels (U/mL) with *HLA-G* alleles in women with an uncomplicated obstetrical history (*n* = 72). Results are from Mann–Whitney U tests.

*HLA-G* Allele	*n*	sHLA-G (U/mL)				
		Me	Min	Max	Q25	Q75
105N	3	6.10	3.80	16.30	3.80	16.30
106	5	6.60	1.00	10.80	2.00	7.80
10101	65	15.40	1.00	257.40	9.70	35.70
10102	42	10.75	2.00	269.60	7.00	25.20
10103	8	11.00	2.00	29.90	5.55	16.35
10106	6	10.90	2.70	41.50	4.20	14.00
10108	5	12.20	2.20	36.10	9.10	36.10
10110	1	18.60	18.60	18.60	18.60	18.60
10401	7	16.70	2.00	147.10	2.70	51.70
10402	1	20.80	20.80	20.80	20.80	20.80
10403	1	9.00	9.00	9.00	9.00	9.00
Total	144	12.30	1.00	269.60	6.80	28.95

**Table 2:** Median (Me), minimum (min), and maximum (max) serum sHLA-G levels (U/mL) in the women with antiphospholipid syndrome (APS), preeclampsia (PE), intrauterine growth restriction (IUGR), and in the control group (C). Results are from Mann–Whitney U tests.

Group	*n*	sHLA-G (U/mL)				
		Me	Min	Max	Q25	Q75
APS; *n* = 57	57	11.90	1.00	458.00	8.00	22.20
PE; *n* = 28	28	10.10	1.70	100.20	5.25	14.55
IUGR; *n* = 18	18	14.90	2.40	160.00	4.60	37.70
C; *n* = 72	72	12.30	1.00	269.60	6.80	28.95
Total	175	11.40	1.00	458.00	6.60	23.40

**Table 3:** The frequency of the *HLA-G* −725 C>G polymorphism in the parental pairs with antiphospholipid syndrome (APS), preeclampsia (PE), intrauterine growth restriction (IUGR), recurrent spontaneous abortion (RSA), and in the control group. Results are from Fisher’s two-sided exact tests.

**The −725 C>G Polymorphism**										
**Polymorphism in Women**	**APS *n* = 70**		**PE *n* = 48**		**IUGR *n* = 34**		**RSA *n* = 58**		**C *n* = 89**	
	***n***	**%**	***n***	**%**	***n***	**%**	***n***	**%**	***n***	**%**
C/G	21	30.00	14	29.17	10	29.41	14	20.69	19	21.35
C/C	47	67.14	34	70.83	24	70.59	46	79.31	69	77.53
G/G	2	2.86	0	0.00	0	0.00	0	0.00	1	1.12
**Polymorphism in Men**	**APS *n* = 54**		**PE *n* = 43**		**IUGR *n* = 35**		**RSA *n* = 58**		**C *n* = 86**	
	***n***	**%**	***n***	**%**	***n***	**%**	***n***	**%**	***n***	**%**
C/G	15	27.78	14	32.56	13	37.14	12	20.69	22	25.58
C/C	38	70.37	28	65.12	22	62.86	45	77.59	63	73.26
G/G	1	1.85	1	2.33	0	0.00	1	1.72	1	1.16

**Table 4:** The frequency of the alleles of the −725 C>G polymorphism in the *HLA-G* promoter region shared by both parents in the pairs with pregnancies complicated by antiphospholipid syndrome (APS), preeclampsia (PE), intrauterine growth restriction (IUGR), recurrent spontaneous abortion (RSA), and the control group. Results are from chi-square tests.

Allele	Allele Shared by Both Partners	APS *n* = 53		PE *n* = 39		IUGR *n* = 32		RSA *n* = 58		C *n* = 80	
		*n*	%	*n*	%	*n*	%	*n*	%	*n*	%
the HLA-G −725 C>G polymorphism	none	1	1.89	1	2.56	0	0.00	36	62.07	1	1.25
	one partner	21	39.62	11	28.21	14	43.75	18	31.03	28	35.00
	both partners	31	58.49	27	69.23	18	56.25	4	6.90	51	63.75
